# The impact of carotid plaque presence and morphology on mortality outcome in cardiological patients

**DOI:** 10.1186/1476-7120-4-16

**Published:** 2006-03-24

**Authors:** Christina Petersen, Patricia B Peçanha, Lucia Venneri, Emilio Pasanisi, Lorenza Pratali, Eugenio Picano

**Affiliations:** 1 CNR, Institute of Clinical Physiology, Pisa, Italy

## Abstract

**Background:**

Carotid plaque severity and morphology can affect cardiovascular prognosis.

We evaluate both the importance of echographically assessed carotid artery plaque geometry and morphology as predictors of death in hospitalised cardiological patients.

**Methods:**

541 hospitalised patients admitted in a cardiological division (age = 66 ± 11 years, 411 men), have been studied through ultrasound Duplex carotid scan and successively followed-up for a median of 34 months. Echo evaluation assessed plaque severity and morphology (presence of heterogeneity and profile).

**Results:**

361 patients showed carotid stenosis (67% with <50% stenosis, 18% with 50–69% stenosis, 9% with >70% stenosis, 4% with near occlusion and 2% with total occlusion). During the follow-up period, there were 83 all-cause deaths (15% of the total population). Using Cox's proportional hazard model, age (RR 1.06, 95% CI 1.03–1.09, p = 0.000), ejection fraction > 50% (RR = 0.62, 95% CI 0.4–0.96, p = 0.03), treatment with statins (RR = 0.52, 95% CI 0.29–0.95, p = 0.34) and the presence of a heterogeneous plaque (RR 1.6; 95% CI, 1.2 to 2.14, p = 0.002) were independent predictors of death. Kaplan – Meier survival estimates have shown the best outcome in patients without plaque, intermediate in patients with homogeneous plaques and the worst outcome in patients with heterogeneous plaques (90% vs 79% vs 73%, p = 0.0001).

**Conclusion:**

In hospitalised cardiological patients, carotid plaque presence and morphology assessed by ultrasound are independent predictors of death.

## Background

Non-invasive carotid artery ultrasound is a well established and valid method which allows to both visualize and quantify atherosclerotic lesions. Ultrasound and autopsy studies have shown that the presence and extent of carotid atherosclerosis correlates with atherosclerosis elsewhere in the circulation, including coronary arteries [1-4]. Several studies have found that the presence of carotid stenosis is a strong predictor of death in the general population [5,6].

Moreover, there is evidence that ultrasonographic B-mode characterization of plaque morphology may be useful in the assessment of the vulnerability of the atherosclerotic lesions [7-10].

Atherosclerotic plaque composition appears to be more important than plaque size in determining adverse events [11]. In particular, lipids and haemorrhages are associated with a more active plaque [12,13], which appear to be more vulnerable to rupture. Ultrasonically assessed plaque morphology provides an insight into the plaque composition and structure as shown by both in vitro [14-18] and in vivo [19-23] studies. Echo-lucent carotid plaques are lipid-rich and have a greater potential for clinical complications [7,10]. Heterogeneous plaques have a hypoechoic component and are associated with the presence of intra-plaque haemorrhage, ulceration and lipids, more likely to result in adverse events [8,21,22].

The purpose of the present study has been to show the importance of echographically assessed carotid artery plaque geometry and morphology as predictors of death in hospitalised cardiological patients.

## Methods

### Patient population

The present study is a single-centre prospective study of the outcome of in-hospital cardiological patients referred to the Cardiology Division of the Institute of Clinical Physiology, CNR, Pisa.

We enrolled 541 in-hospital patients (age = 66 ± 11 years; 411 men) which have been admitted to our cardiological division with a diagnosis, at admission, of: ischemic heart disease (64%), cardiomyopathy (6%), valvular heart disease (8%), arrythymias (9%) and miscellaneous (13%). Patients were followed for a median of 34 months; our end-point was the occurrence of death.

Baseline evaluation included a complete history and physical examination which has been performed by a cardiologist, with information regarding: family history, hypertension (blood pressure ≥ 140/90 mmHg based on the average of repeated readings or patients on antihypertensive drugs), hypercholesterolemia (total cholesterol > 200 mg/dl and/or trigliceridemia > 150 mg/dl or patient on lipid lowering therapy), diabetes mellitus (controlled with diet; oral hypoglycemic agents, or insulin; or fasting glucose level ≥ 126 mg/dl), obesity (body mass index ≥ 30), smoking habits (never smokers; smokers who stopped smoking one month before hospitalisation and current smokers), presence or previous history of coronary artery disease (prevalent angina pectoris, documented previous or current documented acute coronary ischemia or angiographically documented coronary artery stenosis).

### Ultrasound examination

Colour duplex carotid artery scan has been performed by 5 operators with commercially available ultrasound systems (Phillips SONOS 5500, Acuson Sequoia 512, Vingmed System Five). High resolution B-mode, colour Doppler and pulsed-wave Doppler, for both of the carotid arteries, have been performed with an ultrasound linear-array 5 – 10 MHz transducer. Patients have been scanned in a supine position. All of the exams have been stored on s-VHS tapes, recorded from different transducer positions and angles (longitudinal and cross-sectional images) in order to document representative plaque geometry and morphology.

### Assessment of plaque severity

At the initial evaluation, colour duplex scan has been performed on both the right and left common and internal carotid arteries. Categories of carotid stenosis (expressed as the percentage decrease in artery diameter) have been defined on the basis of the B-mode and on velocity criteria: internal carotid artery peak systolic velocity (PSV), end-diastolic velocity (EDV), and *internal carotid artery */common carotid artery PSV ratio. According to the latest consensus for Doppler ultrasound criteria for the diagnosis of internal carotid artery stenosis, the degree of stenosis have been classified [23]. In particular, a non-significant (<50%) stenosis has been identified by a peak systolic velocity <125 cm/sec, and a stenosis ≥ 70% by a peak systolic velocity ≥ 230 cm/sec. In the case of bilateral stenosis or multiple plaques, the carotid plaque with the highest degree of stenosis have been selected for analysis.

### Assessment of plaque morphology

Plaque were defined as a localized protrusion of the vessel wall into the lumen with an area 50% greater than the intima-media thickness of neighbouring sites on visual assessment [24]. Plaque morphology, in terms of echogenicity, were characterized according to a modified version of the classification proposed by Gray- Weale [25]. Plaque echogenicity have been graded into two broad categories: low and high. Low echogenic plaques are black as blood or predominantly black (echolucent), while high echogenic plaques are predominantly white, similar to far-wall adventitia interface (echo-rich or echogenic). Plaques have been classified according to their structural appearance being either heterogeneous or homogeneous. Plaques have been characterized as heterogeneous, if the echogenicity of more than 20% of the plaque differed from the echogenicity of the rest of the plaque by two or more echogenicity grades [26]. We also assessed the plaque surface morphology, classifying it as regular (smooth) or irregular. The plaque surface appearance has been defined as irregular when height variations between 0.4 and 2 mm appeared to be present along the contour of the lesion [27].

### Follow-up data

Follow-up data have been obtained for all of the patients. Total mortality has been the primary end point [28]. Both hospital and physician records, and death certificates, have been used to ascertain the final presentation.

### Statistical analysis

Differences in continuous and categorical variables have been evaluated through the Student t test (two-tailed) for unpaired values and the chi-square test, respectively. Cox proportional-hazard regression model have been performed in order to describe the relationship between the dependent variable (death) and several clinical and echographic parameters, including age, gender, hypertension, diabetes, hypercholesterolemia, plaque morphology and percent stenosis. The Kaplan-Meier method has been used for the survival analysis. All statistical tests have been performed with the SPSS program, version 11.0. Probability values of p < 0.05 have been considered to be significant. The variability between observer has been analysed by using the kappa statistic (κ). κ measures the agreement that occurs above chance and may have values between -1 (complete disagreement) and +1 (perfect agreement). κ values from 0 to 0.20 are categorized as light agreement, those from 0.21 to 0.40 as fair, those from 0.41 to 0.60 as moderate, those from 0.61 to 0.80 as substantial, and those above 0.80 as almost perfect agreement [29].

## Results

361 (67%) of the 541 patients presented a carotid plaque. Baseline clinical characteristics of the patient population separated on the basis of carotid plaque presence, are shown in Table 1. Patients with carotid plaque were older, presented a higher prevalence of diabetes and hypertension and appeared to have a higher prevalence of coronary artery disease and angina at admission.

**Table 1:** Clinical characteristics of patients with and without plaque

	*All (n = 541)*	*Absence of plaque (n = 180)*	*Presence of plaque (n = 361)*	*p*
Age, y	66 ± 10.9	61.7 ± 12.6	68.7 ± 9.2	<0.001
Males	411 (76%)	134 (74.4%)	277 (76.7%)	0.59
Diabetes	112 (20.7%)	28 (15.6%)	84 (23.3%)	0.04
Hypertension	290 (53.6%)	77 (42.8%)	213 (59%)	<0.001
Hypercholesterolemia	310 (57%)	111 (61%)	199 (55%)	0.16
Hypertrigliceridemia	131 (24.2%)	49 (27.2%)	82 (22.7%)	0.28
Obesity	135 (25%)	47 (26.1%)	88 (24.4%)	0.67
Angina at admission	338 (62.5%)	101 (56.1%)	237 (65.7%)	0.03
Ejection fraction <50%	141(26.1%)	35 (19.4%)	106 (29.4%)	0.01
Treatment				
Statins	163 (30%)	49 (27%)	114 (31%)	0.31
Beta-blockers	157 (29%)	48 (26%)	109 (30 %)	0.42
Antiplatelet-agents	376 (70%)	113 (62 %)	263 (73%)	0.018
ACE inhibitors	133 (25%)	32 (17%)	101 (28%)	0.011
Nitrate	323 (60%)	88 (48%)	235 (65%)	0.000

### Ultrasonographic data

The between-observer agreement on plaque morphology has been evaluated separately on a set of 30 consecutive plaques. The inter-observer agreement appeared to be substantial with a κ value of 0.72 (95% CI = 0.53–0.86) for heterogeneity and 0.76 (95% CI 0.56–0.88) for profile. 220 (61%) of the 361 patients with plaque, presented a heterogeneous and 108 ones (30%) an irregular plaque and 75 (20%) presented both criteria. Regarding the stenosis degree, 243 (67.3%) patients presented < 50% stenosis, 66 (18.2%) patients 50–69% stenosis, 32 (8.8%) patients ≥ 70% stenosis, 14 (3.8%) patients presented a near occlusion and 6 (1.6%) patients presented occlusion of carotid artery.

The relative prognostic value of clinical and echographic variables

**Figure 1:**
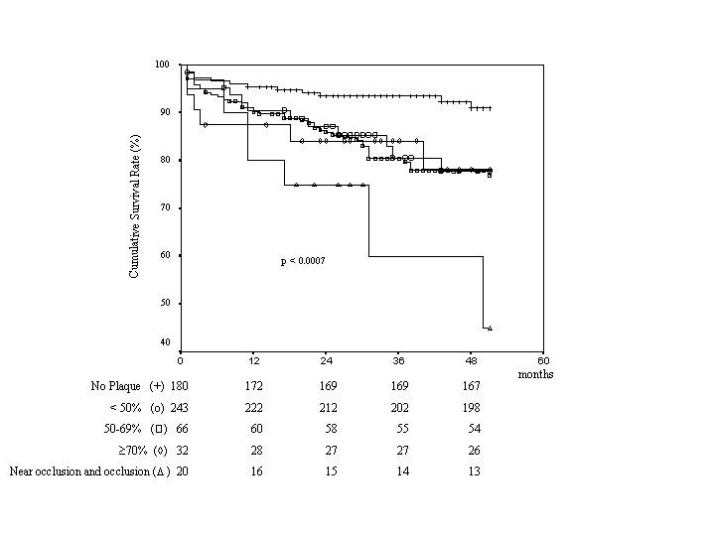
Survival free of event (death) as a function of severity of stenosis. Numbers of patients at risk are listed below each group. Patients without plaque +; < 50% stenosis O; 50–69% stenosis □; ≥ 70% stenosis ◇; near occlusion and occlusion Δ.

**Figure 2:**
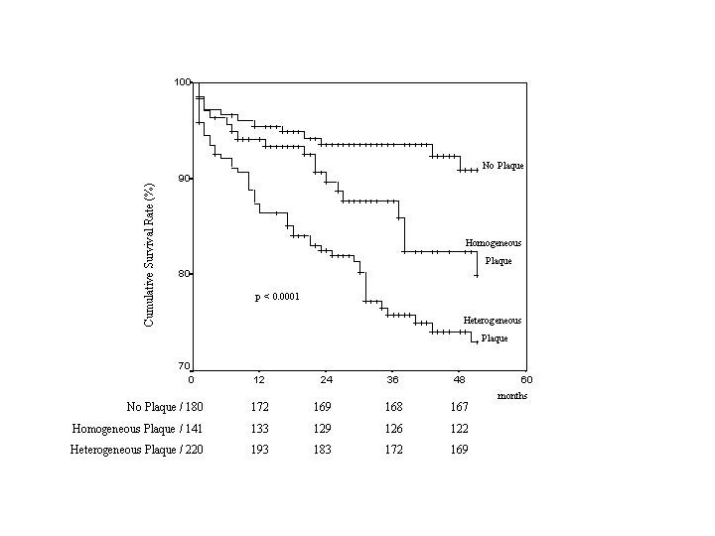
Survival free of event (death) as a function of presence of heterogeneous plaque. Numbers at risk are listed below each group.

Patients were followed for a median of 34 months. During the follow-up period there were 83 all-cause death (15% of the total population); death was attributed to cardiovascular causes in 58 patients (70%). Univariate predictors of total mortality are reported in Table 2. Kaplan-Meier survival estimates for total mortality showed a better outcome for those with no carotid artery stenosis compared to those with the presence of carotid stenosis as a function of stenosis severity, the worst outcome is in the subgroup with near occlusion or occlusion (log rank 19.25, p < 0.0007): Figure 1. With Cox's proportional hazard model analysis independent predictors of death were: age (RR 1.06, 95% CI 1.03–1.09, p = 0.000), and the presence of a heterogeneous plaque (RR 1.6; 95% CI, 1.2 to 2.14, p = 0.002), whereas a normal ejection fraction (RR = 0.62, 95% CI 0.4–0.96, p = 0.03) and treatment with statins (RR = 0.52, 95% CI 0.29–0.95, p = 0.34) were associated with a lower risk for all cause death (Table 3). Kaplan-Meier survival estimates have shown the best outcome in patients without plaque, intermediate outcome in patients with homogeneous plaques, and the worst outcome in patients with heterogeneous plaques (90% vs 79% vs 73%, p = 0.0001) (Figure 2).

**Table 2:** Univariate predictors of death

*Variable*	*p value*	*RR (95% CI)*
Male gender	0.10	0.62 (0.35–1.10)
Age	0.000	1.07 (1.04–1.10)
Coronary artery disease	0.82	0.95 (0.59–1.51)
Ejection fraction >50%	0.009	0.55 (0.35–0.86)
Diabetes	0.19	1.39 (0.84–2.28)
Hypercholesterolemia	0.21	0.70 (0.40–1.17)
Hypertension	0.6	0.89 (0.58–1.37)
Obesity	0.17	0.68 (0.39–1.18)
Presence of Carotid plaque	0.000	2.91 (1.61–5.27)
Heterogeneity	0.000	1.80 (1.36–2.39)
Irregular profile	0.000	1.79 (1.33–2.42)
High degree of stenosis	0.000	1.01 (1.00–1.02)
Treatment		
Statins	0.005	0.45 (0.24–0.77)
Beta-blockers	0.26	0.75 (0.45–1.24)
Antiplatelet-agents	0.43	0.83 (0.52–1.31)
ACE inhibitors	0.65	1.12 (0.69–1.82)
Nitrate	0.17	1.39 (0.87–2.22)

**Table 3:** Multivariate predictors of death

*Variable*	*P*	*RR (95% CI)*
FE > 50%	0.03	0.62 (0.40–0.96)
Age	0.000	1.06 (1.03–1.09)
Heterogeneity	0.002	1.6 (1.20–2.14)
Statins	0.03	0.52 (0.29–0.95)

## Discussion

In hospitalised cardiological patients, carotid plaque presence and morphology assessed by ultrasound are independent predictors of death. For any given level of stenosis heterogeneous and/or hypoechoic plaque texture is associated with a worse outcome, as shown in the carotid [7,8,30,31], coronary [32] and aortic [33] district by transcutaneous, intravascular and transesophageal echocardiography, respectively.

### Plaque histology, biological activity and ultrasound

New understanding of the pathophysiology of atherosclerotic disease has shown that high grade coronary or carotid lesions are not strictly associated with the site of future occlusions [11]. From histopathologic and vascular biologic studies, plaque composition and vulnerability (type of lesion) rather than degree of stenosis (size of lesion), have emerged as crucial factors leading to sudden rupture of the plaque surface, usually with superimposed thrombosis, which underlies the majority of acute occlusions [12]. Homogeneous plaques have been correlated with a fibrous lesion on pathological examination and the heterogeneous plaques have been correlated with the presence of intra-plaque haemorrhage, ulceration and loose stroma containing lipids, cholesterol and proteinaceous deposits [22]. Echo-lucency is associated with lipid-rich plaques [34]. All of the heterogeneous plaques have a echo-lucent component but with B-mode assessment we cannot determine whether echolucent material really represents lipid, haemorrhage, thrombi. A hypo-echoic appearance can also be associated with intra-plaque haemorrhage, which may be the result of intra-plaque neo-vascularization. These small, fragile vessels could represent the underlying anatomic and pathologic changes leading to intramural haemorrhages and rupture. Lipid lakes and intra-plaque haemorrhage are more frequently found in vulnerable plaques, with greater potential for evolution and complication, and are the dominant substrate of hypoechoic and heterogeneous plaques [13].

### Carotid plaque morphology as an index of clinical instability

It is generally agreed that ultrasonic assessment of plaque morphology – in addition to the degree of stenosis – is a predictor of the prognosis, identifying plaques at a higher risk of becoming clinically symptomatic. Subjects with echo-lucent atherosclerotic plaques have increased risk of ischemic cerebrovascular events independently of both degree of stenosis and cardiovascular risk factors [7,31]. Heterogeneous plaques are associated with a higher incidence of cerebrovascular symptoms than homogeneous plaques for all grades of stenosis [8]. The link between echo plaque structure and prognosis do not appear to be limited to the carotid arteries but may apply to virtually all vascular districts where atherosclerotic plaques can be imaged by ultrasound technology. In coronary arteries, echo-lucent zones by intra-vascular ultrasound are also at increased risk of clinical instability [32]. In the ascending thoracic aorta, non-calcified aortic plaques detected by TEE in brain infarction have been associated with a 10-fold increased risk of subsequent events when compared to calcified plaque [33]. Recent studies support the concept that plaque instability is not merely a local vascular incident but rather that plaque instability exists simultaneously at multiple sites of the vascular bed [35]. Honda et al [10] have demonstrated (using radiofrequency analysis) that echo-lucent carotid plaques predict the presence of complex coronary plaques and the development of future coronary complications in stable coronary artery diseased patients. If carotid and coronary artery plaques share common morphological characteristics within individuals, then the ultrasound of the carotid artery may be a simple, non-invasive test to screen asymptomatic subjects at high risk of cardiovascular events.

### Study limitations

Today, ultrasound tissue characterization may be performed by using three methods: subjective assessment (a visual and qualitative analysis of video B-mode image), video densitometry (a quantitative analysis of video image) and acoustic densitometry (a quantitative analysis of unfiltered radio-frequency signal before the video processing chain).

The B-mode assessment can offer a qualitative information about tissue characterization but without precision and with a moderate-to-good agreement between and within-observers [8,26,30,36]. Visual morphological characterization of the carotid plaque correlates well with histological features [37]. This apparently simple method, has been adopted in the present study but it is based on subjective judgment and is operator-dependent.

A videodensitometric [34,38] or – even more – a radiofrequency-based [39-41] approach would have certainly granted a more quantitative, albeit more technologically demanding, approach. Nevertheless, the visual assessment of plaque echogenicity and heterogeneity correlates well with the videodensitometric assessment of gray-scale value and the visual evaluation of carotid plaque remains therefore a valuable method in daily clinical practice [42]. However, there is little doubt that more quantitative, operator-independent criteria would allow the clinician to place the information on plaque morphology on a more solid and reliable basis.

## Conclusion

Ultrasound-based assessment of carotid plaque presence and morphology are independent predictors of death in hospitalised cardiological patients. The present study suggests that the assessment of plaque morphology is more important than plaque size as an indicator of prognosis.

## Competing interests

The author(s) declare that they have no competing interests.

## Authors' contributions

CP participated in the design and coordination of the manuscript, and in the patients enrollment, performed the statistical analysis and wrote the paper. PP participated in the statistical analysis and wrote the paper. LV participated in the patients enrollment and gave critical intellectual suggestions. EmP participated in the patients enrollment and gave critical intellectual suggestions.

LP participated in the patients enrollment and gave critical intellectual suggestions.

E P participated in its design and coordination and was the overall supervisor of the paper. All authors read and approved the final manuscript.
